# Joint Society Statement From the Society for Endocrinology (SfE), the British Thyroid Association (BTA) and the British Association of Endocrine and Thyroid Surgeons (BAETS) Regarding the Association of GLP‐1 Agonists and Thyroid Cancer

**DOI:** 10.1111/cen.70084

**Published:** 2025-12-21

**Authors:** Emma Watts, Jonathan Wadsley, Neil Sharma, Kristien Boelaert, The UK Thyroid Cancer Study Group

**Affiliations:** ^1^ Department of Otolaryngology, Head and Neck Surgery Queen Elizabeth Hospital Birmingham Birmingham UK; ^2^ Department of Applied Health Sciences, College of Medicine and Health University of Birmingham Birmingham UK; ^3^ Department of Oncology Weston Park Cancer Centre Sheffield UK; ^4^ Department of Cancer and Genomic Sciences University of Birmingham Birmingham UK; ^5^ Department of Endocrinology Queen Elizabeth Hospital Birmingham Birmingham UK

Glucagon‐like peptide‐1 receptor agonists (GLP‐1 agonists) have transformed the management of type 2 diabetes and obesity, providing substantial benefits for glycaemic control, weight reduction and cardiometabolic health [1, 2, 3, 4]. Their use is rapidly expanding across both general and specialist practices, with an increasing number of patients also accessing these agents privately for weight management [5, 6]. However, ongoing concern has been raised about a potential association between GLP‐1 agonists and thyroid cancer, in particular medullary thyroid cancer (MTC).

Preclinical rodent studies have found that chronic GLP‐1 agonist exposure stimulates calcitonin secretion, induces C‐cell hyperplasia and increases the incidence of MTC in a dose‐ and drug‐dependent manner [7, 8]. Based on this preclinical data, the US Food and Drug Administration has issued a boxed warning, contraindicating the use of GLP‐1 agonists in those with a personal or family history of MTC or Multiple Endocrine Neoplasia Type 2 (MEN 2) [9]. However, these findings have not been replicated in primates or humans, whose C‐cells exhibit minimal GLP‐1 receptor activity [10, 11, 12, 13, 14].

Clinical evidence remains mixed, but increasingly reassuring. Importantly, no randomised controlled trial in humans has demonstrated a consistent or statistically robust increased incidence of thyroid cancer associated with GLP‐1 receptor agonist therapy [15, 16]. Some pharmacovigilance reports and observational studies have suggested a moderately elevated risk of thyroid cancer [14, 17, 18, 19, 20]. However, large population‐based studies and meta‐analyses found no significant association between GLP‐1 agonist use and thyroid cancer [15, 16, 21, 22, 23, 24, 25, 26, 27, 28]. A recent international meta‐analysis of 98,147 patients found no overall excess risk of thyroid cancer in GLP‐1 agonist users compared with patients treated with Dipeptidyl Peptidase‐4 (DPP‐4) inhibitors (adjusted HR: 0.81, 95% CI 0.59−1.12) [22]. However, long‐term follow‐up is still required. It is also notable that both obesity and diabetes independently increase thyroid cancer risk, complicating the interpretation of observational data [29, 30].

Robust data examining the safety of GLP‐1 agonists in patients who have previously been treated for thyroid cancer is limited. For differentiated thyroid cancer (including papillary, follicular and oncocytic subtypes), GLP‐1 agonists may be used alongside standard oncological surveillance where clinically indicated. A 2022 systematic review found no increased risk of hyperthyroidism, hypothyroidism, thyroiditis, nodules or goitre with GLP‐1 agonists when compared with controls [16]. However, use of GLP‐1 agonists should be avoided in patients with a history of MTC, known pathogenic germline RET variants or a family history of MEN2. For this patient subgroup, initiation of GLP‐1 agonists should only be considered in exceptional cases within a multidisciplinary (MDT) setting. A statement for patients has also been prepared, included as an appendix, to support clear communication and shared decision‐making between clinicians and individuals considering or currently using GLP‐1 agonists (Appendix S1).

Overall, current available evidence suggests no indication of a clinically meaningful short‐term increase in thyroid cancer risk following GLP‐1 agonist initiation. In October 2023, the European Medicines Agency (EMA)'s safety committee Pharmacovigilance Risk Assessment Committee (PRAC) concluded that ‘the available evidence does not support a causal association between [GLP‐1] receptor agonists… and cancer of the thyroid’ [31]. The benefits of therapy are likely to outweigh any potential thyroid‐related risks for most patients. Routine calcitonin measurement or thyroid ultrasound screening is not recommended in unselected patients. Ongoing long‐term surveillance studies will be essential to fully clarify potential late effects.

## Funding

The authors received no specific funding for this work.

## Conflicts of Interest

The authors declare no conflicts of interest.

## Supporting information


**Appendix 1:** GLP‐1 Medication and Thyroid Cancer ‐ Information for Patients.

## Data Availability

Data sharing is not applicable to this article, as no data sets were generated or analysed during the current study.
