# Supplementary material for: Joint Society Statement From the Society for Endocrinology (SfE), the British Thyroid Association (BTA) and the British Association of Endocrine and Thyroid Surgeons (BAETS) Regarding the Association of GLP‐1 Agonists and Thyroid Cancer
Source: Clin Endocrinol (Oxf). 2025 Dec 21;104(5):507–9. doi: 10.1111/cen.70084 (PMC13040510; doi:10.1111/cen.70084)
Supplement: Supplementary file 1 — Appendix 1: GLP‐1 Medication and Thyroid Cancer ‐ Information for Patients. [file CEN-104-507-s001.docx]

# Appendix 1: GLP-1 Medication and Thyroid Cancer - Information for Patients

**What are GLP-1 agonists?**

GLP-1 agonists are a type of medication used mainly to help people with Type 2 diabetes manage their blood sugar (glucose) levels. Some GLP-1 agonists can also be used to treat [obesity](https://my.clevelandclinic.org/health/diseases/11209-weight-control-and-obesity). Most of these medicines are taken by injection.

Other names for this group of medicines include:

- Glucagon-like peptide-1 agonists
- GLP-1 receptor agonists
- Incretin mimetics
- GLP-1 analogs

Examples of currently available medications include:

- Dulaglutide (Trulicity®)
- [Exenatide](https://my.clevelandclinic.org/health/drugs/20513-exenatide-solution-for-injection) (Byetta®)
- [Liraglutide](https://my.clevelandclinic.org/health/drugs/20558-liraglutide-injection-diabetes) (Victoza®, Saxenda®)
- [Lixisenatide](https://my.clevelandclinic.org/health/drugs/19925-lixisenatide-injection) (Adlyxin®)
- Semaglutide injection (Ozempic®, Wegovy®)
- [Tirzepatide](https://my.clevelandclinic.org/health/drugs/23789-tirzepatide-injection) (Mounjaro®)

The first GLP-1 agonist (Exenatide) was approved by the U.S Food and Drug Administration (FDA) in 2005. Researchers are still studying other potential benefits of these medications.

**Do GLP-1 agonists affect the thyroid?**

When these medications were first developed, studies in animals suggested a possible link with a rare thyroid cancer (medullary thyroid cancer). Since then, several large studies have investigated their effects in human populations. Most recently, a large study of nearly 100,000 patients with diabetes treated with *GLP-1 agonists did not show any increased risk of thyroid cancer* in those treated with GLP-1 agonists, compared with people taking other diabetes medication. Although these studies are reassuring, there are currently no large studies reporting the effect of these medications in patients who have previously been treated for thyroid cancer.

**What does this mean for people with thyroid conditions?**

*Differentiated Thyroid Cancer:*

This is the most common form of thyroid cancer and includes papillary, follicular and oncocytic subtypes. There is no evidence that GLP-1 agonists increase the risk of recurrence in patients with differentiated thyroid cancer. If your doctor thinks you are likely to benefit from these medications, the advantages of treatment usually outweigh any possible risks relating to your thyroid cancer.

*Medullary Thyroid Cancer:*

This is a rare type of thyroid cancer which can sometimes run in families. Given previously raised concerns, it is generally recommended that patients with a history of medullary thyroid cancer, those with a family history Multiple Endocrine Neoplasia (MEN) type 2 and those with a known mutation in the RET gene, avoid GLP-1 agonists, unless there is a very strong medical reason to use them.

*Other Thyroid Conditions:*

This includes patients with overactive or underactive thyroid glands, benign nodules or goitres. GLP-1 agonists are considered safe in these patient groups and can be used without concern.

**Summary:**

GLP-1 agonists are safe and effective for most people, including those with common thyroid problems. If you have a history of thyroid cancer, especially medullary thyroid cancer, discuss the risks and benefits carefully with your doctor before starting treatment.
